# Morphological and phylogenetic analyses reveal two new species of *Chaetosphaeriaceae* from Jiangxi Province, China

**DOI:** 10.3897/mycokeys.140.205743

**Published:** 2026-09-16

**Authors:** Weishuai Yu, Xueyuan Wang, Dayu Wu, Hongfeng Wang, Xuefeng Yuan, Yueming Wu

**Affiliations:** 1 National Collection of Plant Protection Microbes (Shandong), College of Plant Protection, Shandong Agricultural University, Taian, Shandong, 271018, China National Collection of Plant Protection Microbes (Shandong), College of Plant Protection, Shandong Agricultural University Taian China; 2 Department of Visual Communication Design, Yeungnam University, Gyeongsan, Gyeongbuk, 38541, Republic of Korea Department of Visual Communication Design, Yeungnam University Gyeongsan Republic of Korea; 3 State Key Laboratory of Agricultural Microbiology, Huazhong Agricultural University, Wuhan, Hubei, 430070, China State Key Laboratory of Agricultural Microbiology, Huazhong Agricultural University Wuhan China

**Keywords:** *
Chloridium
*, morphology, new taxa, *
Paragongromeriza
*, phylogeny

## Abstract

*Chaetosphaeriaceae* is a group of micro-ascomycetes widely distributed in diverse ecosystems worldwide. Group members are commonly found on decaying wood, leaf litter and in moist soil. However, their diversity in soil habitats, especially in understudied bamboo forest ecosystems, remains poorly characterised. In this study, we isolated chaetosphaeriaceous fungi from soil samples collected from a bamboo forest on Xiangyun Mountain, Jiangxi Province, China. We determined their taxonomic placement using maximum likelihood (ML) and Bayesian inference (BI) analyses of concatenated sequence data from three nuclear loci: the internal transcribed spacer (ITS), the large subunit (LSU), and the translation elongation factor 1-α (*tef*1-α). Our results confirm that these isolates belong to two new species: *Chloridium jiangxiense* and *Paragongromeriza jiangxiensis*. We present detailed descriptions and micrographs of both species and compare them with other members of *Chaetosphaeriaceae*. Our findings not only expand the documented species richness of *Chaetosphaeriaceae* in Asia but also underscore the importance of bamboo forest soils as a promising niche for discovering previously unrecognised fungal diversity. Detailed descriptions, photomicrographs, and comparisons with phenotypically similar taxa are provided to facilitate future identification and ecological studies.

## Introduction

Réblová et al. (1999) first validly established the family *Chaetosphaeriaceae*, which includes the type genus *Chaetosphaeria* and six closely related genera: *Ascocodinaea*, *Melanochaeta*, *Melanopsammella*, *Porosphaerella*, *Porosphaerellopsis* and *Striatosphaeria*. In recent decades, the number of recognised genera within this family has fluctuated considerably across taxonomic treatments. Lin et al. (2019) recognized 49 genera (with three considered uncertain), Hyde et al. (2020) accepted 43, Wijayawardene et al. (2022) listed 52 in their 2021 outline, and most recently, Wu and Diao (2022) raised the total to 89, which includes 22 newly described and 10 newly assigned genera. Currently, *Chaetosphaeriaceae* encompasses more than 100 genera (Wu and Diao 2022; Fryar et al. 2023; Réblová and Nekvindová 2023; Hyde et al. 2024).

*Chaetosphaeriaceae (Sordariomycetes)* is a group of micro-ascomycetes widely distributed in diverse ecosystems worldwide. They are commonly found on decaying wood, leaf litter and in moist soil, where they play a key role in lignocellulose degradation and nutrient cycling (Liu et al. 2016; Luo et al. 2016; Perera et al. 2016; Lin et al. 2019; Calabon et al. 2022; Wu and Diao 2022; Zhang et al. 2022; Réblová and Nekvindová 2023). Despite the global increase in *Chaetosphaeriaceae* diversity, systematic surveys in China—particularly in soil microhabitats—remain scarce (Réblová and Nekvindová 2023; Zhang et al. 2024; Réblová et al. 2024). Bamboo forest soils, widespread in southern China and ecologically distinct, have been largely overlooked. Moreover, the species-poor genera *Chloridium* and *Paragongromeriza* are poorly characterised with respect to true species richness and distribution in Asia, hindering both biogeographic understanding and ecological assessments. The present study focuses on the genera *Chloridium* and *Paragongromeriza* within *Chaetosphaeriaceae*, which exhibit distinct morphological characteristics and comprise only a few species.

*Chloridium* was established to classify a group of soil- and wood-inhabiting dematiaceous hyphomycetes that possess phialidic conidiogenous cells with multiple enteroblastic conidiogenous loci (Réblová et al. 2022; Réblová and Nekvindová 2023). It was first introduced by Link (1809), with the hyphomycetous species *Chloridium viride* (currently *Chloridium virescens*) designated as the type species. Réblová et al. (1999) suggested that *Melanopsammella* is the sexual morph of *Chloridium* sensu stricto, *Gonytrichum* and *Chloridium preussii*. Later, Réblová et al. (2016) proposed that *Chloridium*, *Gonytrichum*, and *Melanopsammella* are synonyms, a notion supported by Hyde et al. (2020). Using a polyphasic approach, Réblová et al. (2022) defined *Chloridium* as a monophyletic genus divided into eight sections. It includes 37 species, mainly isolated as saprobes from decaying plants or soil in freshwater and terrestrial habitats, predominantly in moist environments (Réblová et al. 2022; Hyde et al. 2024). Only one uncharacterised *Chloridium* species (*Chloridium* sp.) has been reported in peat swamp forests (Pinnoi et al. 2006; Karimi et al. 2025). *Chloridium* is characterised by phialidic dematiaceous hyphomycetes producing conidia on multiple enteroblastic conidiogenous loci in a sympodial pattern on a central dome within the collarette; the conidia are hyaline or pale brown, aseptate, ellipsoidal, sub-globose, obclavate, occasionally deltoid and adhere in slimy heads or cirrhi (Réblová et al. 2022; Réblová and Nekvindová 2023).

*Paragongromeriza* was originally defined based on its ascospore morphology, which resembles that of *Gongromeriza*, which is typically fusiform to ellipsoidal and septate. However, it is distinguished by differences in ascomatal setae and conidiogenous structures (Réblová et al. 2022; Zhang et al. 2024). *Paragongromeriza* is closely related to the genera *Gongromeriza*, *Phaeostalagmus*, *Sporendocladia* and *Chaetosphaeria* (Réblová et al. 2022; Wu and Diao 2022; Réblová and Nekvindová 2023). Members of *Paragongromeriza* can be differentiated from other taxa in the family by unique features, notably the lack of a sexual morph. They also have conidiogenous cells with apical collarettes (Wei et al. 2018; Zhang et al. 2024). *Paragongromeriza* produces lateral conidia that are either sessile or on short pegs. Most known species have been reported from humus layers in tropical and subtropical Asian forests, with ascomata typically covered by elongated, dark setae, and ascospores ranging from hyaline to pale brown (Réblová et al. 2021; Réblová et al. 2022; Zhang et al. 2024). Currently, only one species (*P. sinensis*) has been documented globally, indicating that this group may be regionally distributed or potentially an undersampled rare fungal taxon (Zhang et al. 2024).

This study aimed to investigate the diversity of *Chaetosphaeriaceae* in soil from bamboo forests in Jiangxi Province, China, with a particular focus on the genera *Chloridium* and *Paragongromeriza*. Using multi-locus (internal transcribed spacer (ITS), LSU, *tef*1-α) phylogenetic analyses, combined with detailed morphological characterisation, the taxonomic placement of these isolates was confirmed. This work is intended to expand the known species inventory of *Chaetosphaeriaceae* in subtropical Asia and to highlight the potential of bamboo forest soils as underexplored reservoirs of fungal diversity.

## Materials and methods

### Strain isolation and preservation

A total of 30 soil samples were collected from a bamboo forest on Xiangyun Mountain, Wuyuan County, Jiangxi Province, China, in May 2024. To ensure representativeness, sampling sites were selected to cover three elevational gradients, with ten sampling points randomly established within each gradient. At each point, surface litter was carefully removed, and a soil core (10 cm in depth) was collected using a soil auger. All samples were individually sealed in sterile plastic bags; collection details were noted (Rathnayaka et al. 2025), taken to the laboratory in an insulated box with ice packs, and stored at 4 °C prior to processing. Detailed records were kept for each sample. The dilution plate method was employed for isolation (Warcup 1950; Abouamama et al. 2023). A 10-g fresh soil sample was weighed and added to a conical flask containing 90 mL of sterile water. The flask was shaken on a rotary shaker at 25 °C and 200 rpm for 30 min to prepare a 10^-1^ soil suspension. Serial tenfold dilutions were prepared, and aliquots of 10^-2^, 10^-3^, and 10^-4^ dilutions were pipetted onto potato dextrose agar (PDA) plates. The inoculum was evenly spread across the plate surface, and the plates were incubated in darkness at 25 °C in a constant temperature incubator. Colony growth was monitored daily; mycelia from the margins of single, distinct, isolated colonies were transferred to new PDA plates using sterile inoculation needles. This process was repeated until pure cultures free from bacterial contamination were obtained. These strains were preserved in 15% sterilised glycerol and stored at 4 °C for future studies. Voucher specimens are housed in the Herbarium of the Department of Plant Pathology, Shandong Agricultural University, Tai’an, China (**HSAUP**). Fungal cultures are stored in the National Collection of Plant Protection Microbes (Shandong) (**NCPMSD**) and the Agricultural Culture Collection of China (**ACCC**). Taxonomic data have been deposited in the Fungal Names repository (https://nmdc.cn/fungalnames/).

### Morphological characterisation

All isolates were inoculated on PDA medium. The isolates’ colony morphology, pigmentation and growth rates were recorded, and PDA plate surfaces (obverse and reverse) were imaged after 14 days using a Canon PowerShot G7X digital camera (Canon, Tokyo, Japan). Microstructures were observed using a stereomicroscope (Olympus SZ61, Olympus Corporation, Tokyo, Japan) and a microscope (Olympus BX53, Olympus Corporation) equipped with differential interference contrast optics. Microscopic fungal structures were captured using BioHD-A20c colour digital cameras (FluoCa Scientific, Shanghai, China) mounted on stereomicroscopes and microscopes. Microstructures were measured randomly using Digimizer v5.6.0 (https://www.digimizer.com), and their mean size was calculated.

### DNA extraction, amplification and sequencing

Pure cultures were grown on PDA medium at 25 °C for 14 days. Fungal genomic DNA was extracted using a Fungal Genomic DNA Extraction Kit (BioTeke, Beijing, China) following the manufacturer’s instructions. The extracted DNA was used in polymerase chain reaction (PCR) targeting the ITS region, large subunit of ribosomal DNA (LSU) and partial translation elongation factor 1-alpha (*tef*1-α) gene. For PCR amplification, the primer pairs ITS5/ITS4, LR0R/LR5 and EF1-983F/EF1-2218R were employed (Table 1). PCR reactions, in a total volume of 50 µl, included 2 µl of DNA template, 2 µl of each primer (forward and reverse; 10 µM each), 25 µl of 2× Taq Master Mix (Vazyme Biotech, Nanjing, China) and 19 µl of distilled deionised water. PCR products were analysed via electrophoresis on a 1% agarose gel stained with Safe Red (Cat. Nos. RM02852 and RM19009, ABclonal Biotechnology Co., Ltd., Wuhan, China) and visualised under ultraviolet light (Ai et al. 2025; Zhang et al. 2025c). Purification and sequencing of the PCR products were performed by Sangon Biotech (Shanghai, China). Sequence data were analysed using MEGA v.7.0 (Kumar et al. 2016). The nucleotide sequences were submitted to the NCBI’s GenBank nucleotide database (https://www.ncbi.nlm.nih.gov/), with their accession numbers listed in Suppl. material 1.

**Table 1.** Loci, PCR primers, and amplification conditions for the molecular markers used in this study.

**Loci**	**PCR primers**	**Sequences (5'→3')**	**PCR cycles**	**References**
ITS	ITS5	GGA AGT AAA AGT CGT AAC AAG G	95 °C 5 min; (95 °C 15 s, 55 °C 15 s, 72 °C 50 s) × 35 cycles; 72 °C 5 min	White et al. (1990)
	ITS4	TCC TCC GCT TAT TGA TAT GC		
LSU	LR0R	GTA CCC GCT GAA CTT AAG C	95 °C 5 min; (95 °C 15 s, 55 °C 15 s, 72 °C 60 s) × 35 cycles; 72 °C 5 min	Vilgalys and Hester (1990)
	LR5	TCC TGA GGG AAA CTT CG		
*tef*1-α	EF1-983F	GCY CCY GGH CAY CGT GAY TTY AT	95 °C 5 min; (95 °C 15 s, 58 °C 15 s, 72 °C 60 s) × 35 cycles; 72 °C 5 min	Rehner and Buckley (2005)
	EF1-2218R	ATG ACA CCR ACR GCR ACR GTY TG		

### Phylogenetic analyses

**Figure 1.**
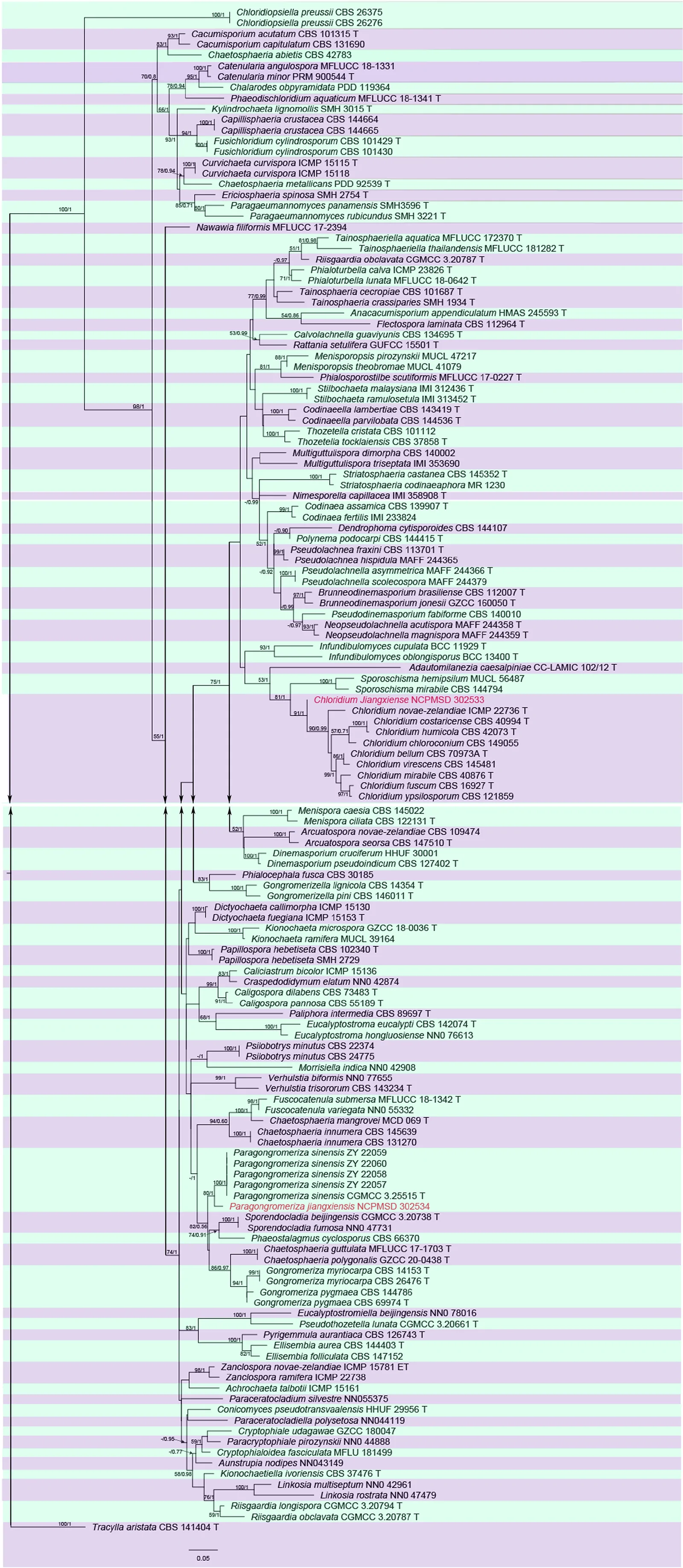
Phylogenetic tree from maximum likelihood analysis using ITS, LSU and *tef*1-α sequence data from *Chaetosphaeriaceae*. Bootstrap support values are ML ≥ 70% and BI ≥ 0.90. *Tracylla aristata* (CBS 141404) served as the outgroup. ‘ET, T’ marks type strains. Strains isolated in the present study are indicated in red.

All sequences were first aligned using the MAFFT v.7 online tool (http://mafft.cbrc.jp/alignment/server/) and then manually corrected in MEGA v. 7.0 (Katoh et al. 2019). The sequences submitted to GenBank did not contain protein-coding genes. The concatenated ITS, LSU and *tef*1-α sequences were used for maximum likelihood (ML; performed on RAxML-HPC2 on XSEDE v.8.2.12) and Bayesian inference (BI; performed on Linux using MrBayes v.3.2.7a with 16 threads) analyses (Zhang et al. 2024). ML analysis used 1000 rapid bootstrap replicates with the GTR+G+I model and default settings. BI analysis employed a fast bootstrap algorithm with automatic stopping, and MrModeltest v.2.3 was used to select the best model for each partition (Nylander 2004; Zhang et al. 2024). The GTR+I+G model, incorporating ITS, LSU and *tef*1-α, was ultimately selected for the analyses. BI posterior probabilities were estimated using Markov Chain Monte Carlo sampling, including four parallel runs of 5,000,000 generations, a sampling frequency of 50 generations and a burn-in fraction of 0.25. Posterior probabilities were derived from the remaining trees. The phylogenetic tree was visualised and optimised using FigTree v.1.4.4 (http://tree.bio.ed.ac.uk/software/figtree), and the final layout was refined in Adobe Illustrator CS6. Isolates from this study are highlighted in red in the phylogenetic tree (Fig. 1).

## Results

### Phylogenetic analyses

Using ITS sequence data, two strains were initially identified as belonging to *Chloridium* and *Paragongromeriza*. A combined phylogenetic analysis of ITS, LSU and *tef*1-α gene regions was then conducted using ML and BI to accurately determine their positions in the phylogenetic tree. The dataset comprised 381 sequences from 142 species, with *Tracylla aristata* (CBS 141404) serving as the outgroup. In total, 2,638 characters, including gaps, were obtained across the regions: ITS: 1–737; LSU: 738–1,704; *tef*1-α: 1,705–2,638. Of these, 1,452 characters were variable, and 1,087 were parsimony-informative. The best-fit nucleotide substitution models selected by MrModeltest for each partition were GTR+I+G for ITS, LSU, and *tef*1-α, respectively. The optimised ML was −8423.5281. Bayesian analysis ran for 1,035,000 generations, producing 1,554 trees, of which 1,399 were used to estimate posterior probabilities. The ML tree confirmed the BI tree topologies; therefore, the ML tree is presented in Fig. 1 [support values include ML bootstrap support (≥70%) and Bayesian posterior probabilities (≥0.90), shown in the first and second positions above nodes, respectively]. Combining morphological and molecular phylogenetic data resulted in the identification of the two strains.

### Taxonomy

#### Chloridium jiangxiense

Taxon classificationFungiChaetosphaerialesChaetosphaeriaceae

W.S. Yu & Y.M. Wu sp. nov.

937F6925-345B-5E8E-9023-BABDD29B3200

Fungal Names: FN 573913

Fig. 2

##### Etymology.

The specific epithet jiangxiense denotes the type locality, Jiangxi Province.

**Figure 2.**
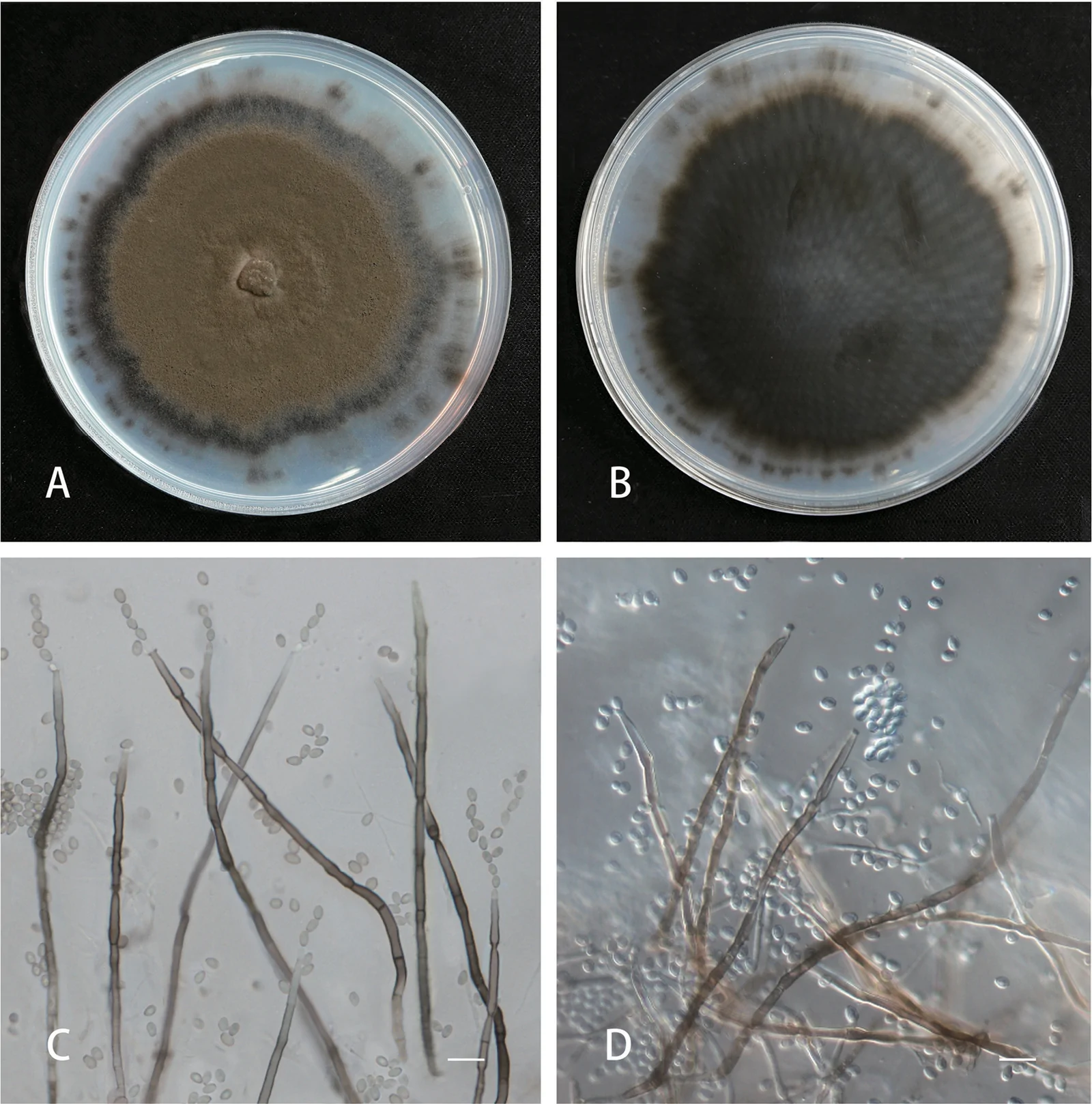
*Chloridium jiangxiense* (NCPMSD 302533). **A, B**. Upper and reverse views of cultures on PDA after 14 days of inoculation at 25 °C; **C, D**. Conidiophores, conidiogenous cells, separating cells and conidia. Scale bars: 25 μm (**A–D**).

##### Holotype.

HSAUP II _26_ 2601.

##### Description.

**Sexual morph**: Undetermined. **Asexual morph**: Mycelium partly superficial, partly immersed. ***Hyphae*** smooth, septate, branched, pale brown, 2–3-μm wide (number of measurements (n) = 30, mean = 2.4 μm). ***Conidiophores*** lateral or terminal, solitary, erect, septate, pale brown, smooth, 50–150-μm long (n = 30, mean = 95.6 μm), percurrenty proliferating. ***Conidiogenous cells*** terminal, phiallidic, solitary, subcylindrical, tapering towards apex, with neck below the collarette distinctly constricted, pale brown, smooth, 20–60 × 2–3 μm (n = 30, mean = 38.5 × 2.5 μm), percurrently proliferating one to several times through previous collarette; collarette conspicuous, funnel-shaped, slightly darker. ***Conidia*** aseptate, ellipsoidal, with a truncate hilum, round at apex, pale yellow, smooth, with 1–2 guttules, 5.0–7.0 × 3.0–4.5 μm (n = 50, mean = 6.1 × 3.7 μm), often remaining in loose linear chains.

##### Culture characteristics.

Colonies cultured on PDA at 25 °C in darkness, reaching 3–4 cm in diameter in 2 weeks. Dark grey to dark brown, often with concentric rings.

##### Material examined.

• China, Jiangxi Province, Shangrao City, Wuyuan County, Xiangyun Mountain, on bamboo grove soil, 29^th^ May 2024, Y.M. Wu, (holotype, HSAUP II _26_ 2601), ex-type ACCC 34269 = NCPMSD 302533.

##### GenBank accession numbers.

NCPMSD 302533: ITS = PZ412717, LSU = PZ412718, *tef*1-α = PZ418266.

##### Notes.

Based on phylogenetic analyses using three gene sequences (ITS, LSU and *tef*1-α), *Chloridium jiangxiense* (NCPMSD 302533) was found to be closely related to *C. novae-zelandiae* (ICMP 22736). However, pairwise sequence comparisons showed that they differ by 84.06% in ITS, 89.80% in LSU, and 91.81% in *tef*1-α, indicating substantial genetic divergence that supports their recognition as distinct species. Compared with the phylogenetically related *C. novae-zelandiae*, the new species differs markedly in morphology (Réblová et al. 2022). *Chloridium novae-zelandiae* is known primarily from its sexual morph found on natural substrate, producing smaller part-ascospores (4.0–5.5 × 2.5–3.5 μm) and developing a simple, occasionally branched *Chloridium*-type asexual morph in axenic culture, without chlamydospores. In contrast, *C. jiangxiense* consistently forms larger (5.0–7.0 × 3.0–4.5 μm) ellipsoidal conidia with a distinct truncate hilum and typically 1–2 prominent guttules. Additionally, *C. jiangxiense*’s conidiogenous cells are distinct, percurrently proliferating, with a swollen and constricted neck below the collarette, a feature not observed in *C. novae-zelandiae*. Thus, the combined evidence supports *C. jiangxiense* as a new species.

#### Paragongromeriza jiangxiensis

Taxon classificationFungiChaetosphaerialesChaetosphaeriaceae

W.S. Yu & Y.M. Wu sp. nov.

5AB6BD91-237E-5D44-808C-E1E7666F8F7A

Fungal Names: FN 573914

Fig. 3

##### Etymology.

The specific epithet jiangxiensis denotes the type locality, Jiangxi Province.

**Figure 3.**
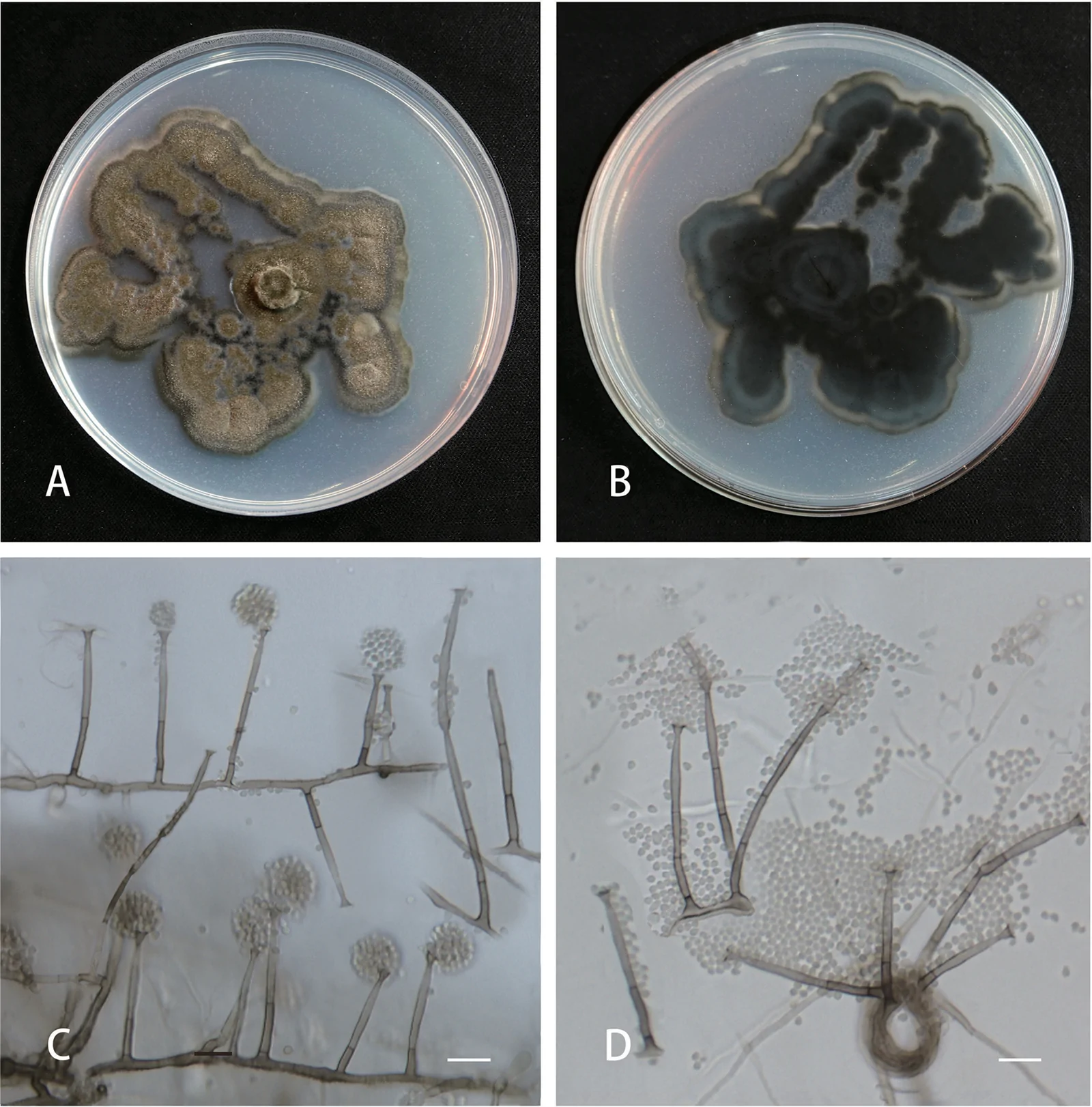
*Paragongromeriza jiangxiensis* (NCPMSD 302534). **A, B**. Upper and reverse views of cultures on PDA after 14 days of inoculation at 25 °C; **C, D**. Conidiophores, conidiogenous cells, separating cells and conidia. Scale bars: 25 μm (**A–D**).

##### Holotype.

HSAUP II _26_ 2602.

##### Description.

**Sexual morph**: Not observed. **Asexual morph**: Mycelium mostly superficial, partly immersed. ***Hyphae*** septate, branched, smooth, olivaceous-brown to pale brown, 1.5–3.0-μm wide (n = 30, mean = 2.2 μm). ***Conidiophores*** well-differentiated, solitary, erect, pale brown, smooth, percurrently proliferating, 20–110-μm long (n = 30, mean = 58.3 μm), 1.5–3.0-μm wide (n = 30, mean = 2.3 μm). ***Conidiogenous cells*** terminal, solitary, subcylindrical, tapering towards apex, with a distinct constriction at the neck below flared collarette, percurrently proliferating once or twice through previous collarette, pale brown, smooth, 15–60 × 1.5–3.0 μm (n = 30, mean = 32.4 × 2.2 μm); collarette conspicuous, funnel-shaped or lageniform, slightly darker. ***Conidia*** unicellular, ellipsoidal or sub-globose, with a truncate base, pale olivaceous-brown, surface slightly roughened, 3.5–5.0 × 3.0–4.0 μm (n = 50, mean = 4.2 × 3.5 μm), often aggregated into a slimy spore mass.

##### Culture characteristics.

Colonies cultured on PDA at 25 °C in darkness, with a faintly radiate texture, grey to dark yellowish-brown; slow-growing, reaching 3–4 cm in diameter in 4 weeks at 25 °C.

##### Material examined.

• China, Jiangxi Province, Shangrao City, Wuyuan County, Xiangyun Mountain, on bamboo grove soil, 29^th^ May 2024, Y.M. Wu, (holotype, HSAUP II _26_ 2602), ex-type culture ACCC 34268 = NCPMSD 302534.

##### GenBank accession numbers.

NCPMSD 302534: ITS = PZ412604, LSU = PZ412711, *tef*1-α = PZ419943.

##### Notes.

Based on phylogenetic analyses using three gene sequences (ITS, LSU and *tef*1-α), *Paragongromeriza jiangxiensis* (NCPMSD 302534) was found closely related to *P. sinensis* (CGMCC 3.25515). Morphologically, *P. jiangxiensis* resembles *P. sinensis* in producing macronematous, solitary percurrently proliferating conidiophores, with terminal collarettes and aseptate conidia (Zhang et al. 2024). However, it differs from *P. sinensis* as follows: chlamydospores are absent in *P. jiangxiensis*, whereas *P. sinensis* forms lateral, sessile or short-stalked chlamydospores; *P. jiangxiensis*’ conidia are slightly roughened, ellipsoidal to sub-globose with a truncate base and relatively large (3.5–5.0 × 3.0–4.0 μm) compared with the smooth, cuneiform to obovate conidia of *P. sinensis*, possessing a broadly rounded base and smaller size (2.0–4.0 × 2.0–2.5 μm); and *P. jiangxiensis*’ conidiophores reach a greater length. These morphological and cultural differences distinguish *P. jiangxiensis* from *P. sinensis*; therefore, we introduce *P. jiangxiensis* as a novel species.

## Discussion

During a survey of soil fungi in Jiangxi Province, southern China, we identified two new species, *C. jiangxiense* and *P. jiangxiensis*. These are described here using morphological characterisation and multi-locus phylogenetic analyses (ITS, LSU and *tef*1-α). Jiangxi is known for its rich fungal diversity, but its soil mycobiota is still poorly documented (Zhang et al. 2023).

In the combined ITS, LSU and *tef*1-α phylogenetic tree, *C. jiangxiense* clustered within *Chloridium* and was closely related to *C. novae-zelandiae* (ICMP 22736). This arrangement aligns with the sectional classification proposed by Réblová et al. (2022); the new species is grouped in a clade defined by phialidic conidiogenous cells with prominent collarettes and hyaline to pale-brown aseptate conidia. *Paragongromeriza jiangxiensis* is identified as a close relative of *P. sinensis* (CGMCC 3.25515) within the *Paragongromeriza* lineage, supported by high bootstrap values. Both species have macronematous, solitary conidiophores with terminal collarettes and aseptate conidia. Nonetheless, *P. jiangxiensis* differs notably by lacking chlamydospores, whereas *P. sinensis* consistently produces lateral, sessile or short-stalked chlamydospores (Zhang et al. 2024). These morphological and cultural differences distinguish the two species.

The phylogenetic evidence supporting our taxonomic conclusions warrants further interpretation. In the phylogeny (Fig. 1), *C. jiangxiense* (NCPMSD 302533) forms a well-supported clade with *C. novae-zelandiae* (ICMP 22736). This sister relationship, while moderately supported by bootstrap, is robustly reinforced by the high pairwise sequence divergence between the two taxa across all three loci. These values substantially exceed the intraspecific variation typically observed within *Chloridium* (Réblová et al. 2022), providing strong molecular evidence for their independent species status. Similarly, *P. jiangxiensis* (NCPMSD 302534) is placed as a close relative of *P. sinensis* (CGMCC 3.25515) with strong statistical support, yet they are distinguished by consistent morphological disparities, including the absence of chlamydospores and larger, slightly roughened conidia. The congruence between phylogenetic clustering and morphological diagnosability fulfils the criteria of the genealogical concordance phylogenetic species recognition (GCPSR) concept (Taylor et al. 2000), reinforcing the recognition of both taxa as novel species. This interpretation moves beyond merely stating the phylogenetic placement and explicitly links the statistical support and character divergence to the taxonomic decisions made.

Both new species were isolated from soil in a bamboo grove on Xiangyun Mountain, Wuyuan County, Jiangxi Province. The habitat is moist, shaded, and rich in organic matter, characteristic of *Chaetosphaeriaceae* members that serve as saprobes involved in lignocellulose degradation (Zhang et al. 2025a, 2025b). The isolation of *C. jiangxiense* and *P. jiangxiensis* from soil, rather than from decaying wood or leaf litter, is consistent with the ecological preference of chaetosphaeriaceous fungi, which are primarily associated with humus-rich soil, particularly members of the *Chloridium* complex (Réblová et al. 2022; Réblová and Nekvindová 2023). This finding further supports the view that humus-containing soil serves as an important reservoir for these fungi and highlights the value of targeting such microhabitats in biodiversity surveys. *Paragongromeriza* species have mainly been reported from humus layers in tropical and subtropical Asian forests (Zhang et al. 2024). The discovery of *P. jiangxiensis* in Jiangxi Province extends the known geographical range of this genus within China and underscores the potential for future soil-focused surveys in southern China to uncover additional hidden diversity.

In the present study, we used the loci ITS, LSU and *tef*1-α, which are standard for species delimitation within *Chaetosphaeriaceae*. However, adding more variable markers, such as RPB2 or β-tubulin, or using genome-wide data, could enhance resolution for closely related species groups, especially in *Chloridium*, where high morphological variability is well documented (Réblová and Nekvindová 2023). The sexual forms of both new species remain undetermined; future research using different culture conditions or co-culturing with potential hosts may stimulate the development of sexual structures, providing additional taxonomic characters.

In conclusion, combined morphological and phylogenetic evidence firmly establishes *C. jiangxiense* and *P. jiangxiensis* as two novel species from soil in Jiangxi Province, southern China. Their discovery broadens our understanding of the diversity of *Chaetosphaeriaceae* and highlights the ecological importance of soil habitats in this region.

## Supplementary Material

XML Treatment for Chloridium jiangxiense

XML Treatment for Paragongromeriza jiangxiensis

## Additional information

### Artificial Intelligence (AI) use

The authors accept full responsibility for the content of the manuscript, including the disclosure of any use of AI.

No AI tools were used in the preparation of this manuscript.

### Author ORCIDs

W. Yu https://orcid.org/0000-0002-9981-4949

X. Wang https://orcid.org/0009-0008-2637-6141

D. Wu https://orcid.org/0009-0000-4066-7801

### Data availability

All of the data that support the findings of this study are available in the main text or Supplementary Information.

## Supplementary materials

Supplementary material 1 GenBank accession numbers Data type docx Explanation note **table SS1**. GenBank accession numbers of the taxa used in phylogenetic reconstruction. https://binary.pensoft.net/file/1776269This dataset is made available under the Open Database License (http://opendatacommons.org/licenses/odbl/1.0/). The Open Database License (ODbL) is a license agreement intended to allow users to freely share, modify, and use this Dataset while maintaining this same freedom for others, provided that the original source and author(s) are credited. Weishuai Yu, Xueyuan Wang, Dayu Wu, Hongfeng Wang, Xuefeng Yuan, Yueming Wu
