# Supplementary material for: Morphological and phylogenetic analyses reveal two new species of Chaetosphaeriaceae from Jiangxi Province, China
Source: MycoKeys. 2026 Sep 16;140:131–46. doi: 10.3897/mycokeys.140.205743 (PMC13601918; doi:10.3897/mycokeys.140.205743)
Supplement: Supplementary file 1 [file mycokeys-140-131-s001.docx]

Table S1. GenBank accession numbers of the taxa used in phylogenetic reconstruction.

| **Taxon** | **Strain** | **GenBank Accession numbers** | | |
| --- | --- | --- | --- | --- |
|  |  | **ITS** | **LSU** | ***TEF1-α*** |
| *Achrochaeta talbotii* | ICMP 15161 | MT454480 | MT454495 | OL653988 |
| *Adautomilanezia caesalpiniae* | CCLAMIC 102/12 T | KX821777 | KU170671 | - |
| *Anacacumisporium appendiculatum* | HMAS 245593 T | KP347129 | KT001553 | - |
| *Arcuatospora novaezelandiae* | CBS 109474 | MW984569 | MW984552 | OL653989 |
| *Arcuatospora seorsa* | CBS 147510 T | MW984572 | MW984555 | OL653990 |
| *Aunstrupia nodipes* | NN043149 | OL627566 | OL655011 | - |
| *Brunneodinemasporium brasiliense* | CBS 112007 T | JQ889272 | JQ889288 | - |
| *Brunneodinemasporium jonesii* | GZCC 160050 T | KY026058 | KY026055 | - |
| *Cacumisporium acutatum* | CBS 101315 T | OR134682 | OR134626 | OR130762 |
| *Cacumisporium capitulatum* | CBS 131690 | OR134684 | OR134628 | OR130764 |
| *Caliciastrum bicolor* | ICMP 15136 | OR134689 | OR134633 | OR130769 |
| *Caligospora dilabens* | CBS 734.83 ET | OR134691 | OR134636 | OR130771 |
| *Caligospora pannosa* | CBS 551.89 T | OR134692 | OR134637 | OR130772 |
| *Calvolachnella guaviyunis* | CBS 134695 T | KJ834524 | KJ834525 | - |
| *Capillisphaeria crustacea* | CBS 144664 | OR134694 | OR134639 | OR130774 |
| *Capillisphaeria crustacea* | CBS 144665 | OR134695 | OR134640 | OR130775 |
| *Catenularia angulospora* | MFLUCC 181331 | MK828638 | MK835840 | MN194089 |
| *Catenularia minor* | PRM 900544 T | MW987827 | MW987822 | OL653993 |
| *Chaetosphaeria abietis* | CBS 427.83 | OR134697 | OR134642 | OR130777 |
| *Chaetosphaeria guttulata* | MFLUCC 171703 T | MK828636 | MK835837 | MN194087 |
| *Chaetosphaeria innumera* | CBS 145639 | OP455358 | OP455464 | OP465036 |
| *Chaetosphaeria innumera* | CBS 131270 | OR134698 | OR134643 | OR130778 |
| *Chaetosphaeria mangrovei* | MCD 069 T | MG813821 | MG813820 | - |
| *Chaetosphaeria metallicans* | PDD 92539 T | EU037893 | EU037899 | - |
| *Chaetosphaeria polygonalis* | GZCC 200438 T | OP377861 | OP377946 | OP473040 |
| *Chalarodes obpyramidata* | PDD 119364 | MW987828 | MW987823 | OL653995 |
| *Chloridiopsiella preussii* | CBS 263.75 | OR134700 | OR134645 | OR130780 |
| *Chloridiopsiella preussii* | CBS 262.76 | OR134701 | OR134646 | OR130781 |
| *Chloridium bellum* | CBS 709.73A T | OP455360 | OP455466 | OP464934 |
| *Chloridium chloroconium* | CBS 149055 | OP455398 | OP455505 | OP464972 |
| *Chloridium costaricense* | CBS 409.94 T | OP455399 | OP455506 | OP464973 |
| *Chloridium fuscum* | CBS 169.27 T a | OP455404 | OP455511 | OP464979 |
| *Chloridium humicola* | CBS 420.73 T | OP455417 | OP455524 | OP464993 |
| ***Chloridium jiangxiense*** | **NCPMSD 302533 T** | **PZ412717** | **PZ412718** | **PZ418266** |
| *Chloridium mirabile* | CBS 408.76 T | OP455420 | OP455526 | OP464994 |
| *Chloridium novae-zelandiae* | ICMP 22736 T | OP455423 | OP455530 | OP464998 |
| *Chloridium virescens* | CBS 145481 | OP455439 | OP455547 | OP465014 |
| *Chloridium ypsilosporum* | CBS 121859 | OP455452 | OP455560 | OP465024 |
| *Codinaea assamica* | CBS 139907 T | OL654077 | OL654134 | OL653997 |
| *Codinaea fertilis* | IMI 233824 | OL654080 | OL654137 | OL654000 |
| *Codinaeella lambertiae* | CBS 143419 T | OL654084 | OL654141 | OL654009 |
| *Codinaeella parvilobata* | CBS 144536 T | OL654100 | OL654157 | OL654027 |
| *Conicomyces pseudotransvaalensis* | HHUF 29956 T | LC001710 | LC001708 | - |
| *Craspedodidymum elatum* | NN042874 | OL627547 | OL655004 | - |
| *Cryptophiale udagawae* | GZCC 180047 | MN104608 | MN104619 | - |
| *Cryptophialoidea fasciculata* | MFLU 181499 | MH758195 | MH758208 | - |
| *Curvichaeta curvispora* | ICMP 15115 T | OR134705 | OR134650 | OR130785 |
| *Curvichaeta curvispora* | ICMP 15118 | OR134706 | OR134651 | OR130786 |
| *Dendrophoma cytisporoides* | CBS 144107 | MT118234 | MT118205 | OL654032 |
| *Dictyochaeta callimorpha* | ICMP 15130 | MT454483 | MT454498 | MT454673 |
| *Dictyochaeta fuegiana* | ICMP 15153 T | MT454487 | EF063574 | MT454677 |
| *Dinemasporium cruciferum* | HHUF 30001 | AB900895 | AB934039 | AB934089 |
| *Dinemasporium pseudoindicum* | CBS 127402 T | JQ889277 | JQ889293 | - |
| *Ellisembia aurea* | CBS 144403 T | MH836375 | MH836376 | - |
| *Ellisembia folliculata* | CBS 147152 | OL654105 | OL654162 | OL654033 |
| *Ericiosphaeria spinosa* | S.M.H. 2754 T | MW984575 | AF466079 | - |
| *Eucalyptostroma eucalypti* | CBS 142074 T | KY173408 | KY173500 | - |
| *Eucalyptostroma hongluosiense* | NN076613 | OL628127 | OL655185 | - |
| *Eucalyptostromiella beijingensis* | NN078016 | OL628501 | OL655251 | - |
| *Flectospora laminata* | CBS 112964 T | MW984576 | MW984558 | OL654034 |
| *Fuscocatenula submersa* | MFLUCC 181342 T | MK828634 | MK835835 | MN194085 |
| *Fuscocatenula variegata* | NN055332 | OL627817 | OL655124 | - |
| *Fusichloridium cylindrosporum* | CBS 101429 ET | OR134709 | OR134653 | OR130789 |
| *Fusichloridium cylindrosporum* | CBS 101430 | OR134710 | OR134654 | OR130790 |
| *Gongromeriza myriocarpa* | CBS 141.53 T | OP455456 | OP455564 | OP465029 |
| *Gongromeriza myriocarpa* | CBS 264.76 NT | OP455457 | OP455565 | OP465028 |
| *Gongromeriza pygmaea* | CBS 144786 | OP455458 | OP455566 | OP465030 |
| *Gongromeriza pygmaea* | CBS 699.74 T | OP455459 | OP455567 | OP465031 |
| *Gongromerizella lignicola* | CBS 143.54 T | OP455460 | OP455568 | OP465032 |
| *Gongromerizella pini* | CBS 146011 T | MT223787 | MT223882 | - |
| *Infundibulomyces cupulatus* | BCC 11929 T | EF113976 | EF113979 | - |
| *Infundibulomyces oblongisporus* | BCC 13400 T | EF113977 | EF113980 | - |
| *Kionochaeta microspora* | GZCC 180036 T | MN104607 | MN104618 | - |
| *Kionochaeta ramifera* | MUCL 39164 | MW144421 | MW144404 | OL654036 |
| *Kionochaetiella ivoriensis* | CBS 374.76 T | MH860988 | MH872758 | - |
| *Kylindrochaeta lignomollis* | S.M.H. 3015 T | EU037896 | AF466073 | - |
| *Linkosia multiseptum* | NN042961 | OL627557 | OL655008 | - |
| *Linkosia rostrata* | NN047479 | OL627662 | OL655059 | - |
| *Menispora caesia* | CBS 145022 | OL654107 | OL654164 | OL654039 |
| *Menispora ciliata* | CBS 122131 T | EU488736 | OL654165 | OL654040 |
| *Menisporopsis pirozynskii* | MUCL 47217 | MW984579 | MW984561 | OL654047 |
| *Menisporopsis theobromae* | MUCL 41079 | MW984580 | MW984562 | OL654048 |
| *Morrisiella indica* | NN042908 | OL627551 | OL655005 | - |
| *Multiguttulispora dimorpha* | CBS 140002 | MW984582 | MW984564 | OL654049 |
| *Multiguttulispora triseptata* | IMI 353690 | MW984584 | MW984566 | OL654050 |
| *Nawawia filiformis* | MFLUCC 172394 | MH758196 | MH758209 | OP473069 |
| *Neopseudolachnella acutispora* | MAFF 244358 T | AB934065 | AB934041 | AB934091 |
| *Neopseudolachnella magnispora* | MAFF 244359 T | AB934066 | AB934042 | AB934092 |
| *Nimesporella capillacea* | IMI 358908 T | OL654114 | OL654171 | OL654051 |
| *Paliphora intermedia* | CBS 896.97 IT | MH862682 | EF204501 | - |
| *Papillospora hebetiseta* | CBS 102340 T | AF178549 | AF178549 | OL653994 |
| *Papillospora hebetiseta* | S.M.H. 2729 | AY906955 | AF466069 | - |
| *Paraceratocladiella polysetosa* | NN044119 | OL627605 | OL655027 | - |
| *Paraceratocladium silvestre* | NN055375 | OL627830 | OL655132 | - |
| *Paracryptophiale pirozynskii* | NN044888 | OL627641 | OL655047 | - |
| *Paragaeumannomyces panamensis* | S.M.H. 3596 T | AY906948 | MT118218 | - |
| *Paragaeumannomyces rubicundus* | S.M.H. 3221 T | MT118242 | MT118224 | - |
| ***Paragongromeriza jiangxiensis*** | **NCPMSD 302534 T** | **PZ412604** | **PZ412711** | **PZ419943** |
| *Paragongromeriza sinensis* | CGMCC 3.25515 T | OR680535 | OR680602 | OR842950 |
| *Paragongromeriza sinensis* | ZY22.057 | OR680536 | OR680603 | OR842951 |
| *Paragongromeriza sinensis* | ZY22.058 | OR680537 | OR680604 | OR842952 |
| *Paragongromeriza sinensis* | ZY22.059 | OR680538 | OR680605 | OR842953 |
| *Paragongromeriza sinensis* | ZY22.060 | OR680539 | OR680606 | OR842954 |
| *Phaeodischloridium aquaticum* | MFLUCC 181341 T | MK828639 | MK835841 | MN194090 |
| *Phaeostalagmus cyclosporus* | CBS 663.70 | MH859892 | MH871680 | - |
| *Phialocephala fusca* | CBS 301.85 | AF486122 | MH873571 | - |
| *Phialogeniculata guadalcanalensis* | MFLUCC 18-0260 T | MK828625 | MK835825 | MN194078 |
| *Phialosporostilbe scutiformis* | MFLUCC 170227 T | MH758194 | MH758207 | - |
| *Phialoturbella calva* | ICMP 23826 T | MW984585 | MW984567 | OL654052 |
| *Phialoturbella lunata* | MFLUCC 180642 T | MK828624 | MK835824 | MN194077 |
| *Polynema podocarpi* | CBS 144415 T | MH327797 | MH327833 | - |
| *Pseudodinemasporium fabiforme* | CBS 140010 | KR611889 | KR611906 | - |
| *Pseudolachnea fraxini* | CBS 113701 T | JQ889287 | JQ889301 | AB934096 |
| *Pseudolachnea hispidula* | MAFF 244365 | AB934072 | AB934048 | AB934098 |
| *Pseudolachnella asymmetrica* | MAFF 244366 T | AB934073 | AB934049 | AB934099 |
| *Pseudolachnella scolecospora* | MAFF 244379 | AB934086 | AB934062 | AB934112 |
| *Pseudothozetella lunata* | CGMCC 3.20661 T | OL628034 | OL655157 | - |
| *Psilobotrys minutus* | CBS 223.74 | OP455462 | OP455570 | OP465034 |
| *Psilobotrys minutus* | CBS 247.75 | OP455463 | OP455571 | OP465035 |
| *Pyrigemmula aurantiaca* | CBS 126743 T | HM241692 | HM241692 | - |
| *Rattania setulifera* | GUFCC 15501 T | GU191794 | HM171322 | - |
| *Riisgaardia longispora* | CGMCC 3.20794 T | OL627701 | OL655085 | - |
| *Riisgaardia obclavata* | CGMCC 3.20787 T | OL627568 | OL655013 | - |
| *Sporendocladia beijingensis* | CGMCC 3.20738 T | OL628290 | OL655217 | - |
| *Sporendocladia fumosa* | NN047731 | OL627669 | OL655065 | - |
| *Sporoschisma hemipsilum* | MUCL 56487 | MW987829 | MW987824 | - |
| *Sporoschisma mirabile* | CBS 144794 | MW987830 | MW987825 | - |
| *Stilbochaeta malaysiana* | IMI 312436 T | OL654121 | OL654178 | OL654059 |
| *Stilbochaeta ramulosetula* | IMI 313452 ET | OL654124 | OL654181 | OL654062 |
| *Striatosphaeria castanea* | CBS 145352 T | MT118244 | MT118229 | - |
| *Striatosphaeria codinaeophora* | M.R. 1230 | AF178546 | AF178546 | - |
| *Tainosphaeria cecropiae* | CBS 101687 T | MW984586 | MW984568 | OL654064 |
| *Tainosphaeria crassiparies* | S.M.H. 1934 T | MW984587 | AF466089 | - |
| *Tainosphaeriella aquatica* | MFLUCC 172370 T | MZ161197 | MZ161195 | MZ170694 |
| *Tainosphaeriella thailandense* | MFLUCC 181282 T | MZ161198 | MZ161196 | MZ170695 |
| *Thozetella cristata* | CBS 101112 | OL654126 | OL654183 | OL654065 |
| *Thozetella tocklaiensis* | CBS 378.58 T | OL654128 | OL654185 | OL654067 |
| *Verhulstia biformis* | NN077655 | OL628434 | OL655237 | - |
| *Verhulstia trisororum* | CBS 143234 T | MG022181 | MG022160 | - |
| *Zanclospora novaezelandiae* | ICMP 15781 ET | MW144429 | MW144411 | MW147330 |
| *Zanclospora ramifera* | ICMP 22738 T | MW144433 | MW144415 | MW147334 |
| *Tracylla aristata* | CBS 141404 ET | OL654129 | OL654186 | OL654068 |

Notes: Species identified in this study are shown in bold. Those marked ‘ET, T’ in the table are ex-type or ex-epitype strains. ‘-’: not available.
